# Tumor-associated macrophages and Tregs influence and represent immune cell infiltration of muscle-invasive bladder cancer and predict prognosis

**DOI:** 10.1186/s12967-023-03949-3

**Published:** 2023-02-15

**Authors:** Florestan J. Koll, Severine Banek, Luis Kluth, Jens Köllermann, Katrin Bankov, Felix K.-H. Chun, Peter J. Wild, Andreas Weigert, Henning Reis

**Affiliations:** 1 Department of Urology, University Hospital Frankfurt, Goethe University Frankfurt, Theodor-Stern-Kai 7, 60590 Frankfurt am Main, Germany; 2 Frankfurt Cancer Institute (FCI), University Hospital, Goethe University Frankfurt, Theodor-Stern-Kai 7, 60590 Frankfurt am Main, Germany; 3 University Cancer Center (UCT) Frankfurt, University Hospital, Goethe University Frankfurt, Theodor-Stern-Kai 7, 60590 Frankfurt am Main, Germany; 4 Institute of Biochemistry I, Faculty of Medicine, Goethe-University Frankfurt, Frankfurt, Germany; 5 Dr. Senckenberg Institute of Pathology, University Hospital Frankfurt, 60590 Frankfurt am Main, Germany; 6 Frankfurt Institute for Advanced Studies, 60438 Frankfurt am Main, Germany

**Keywords:** Bladder cancer, MIBC, TAM, Immune cells, Chemotherapy, Treg

## Abstract

**Introduction and objective:**

Muscle-invasive urothelial bladder cancer (MIBC) is associated with limited response rates to systemic therapy, risk of recurrence and death. Tumor infiltrating immune cells have been associated with outcome and response to chemo-and immunotherapy in MIBC. We aimed to profile the immune cells in the tumor microenvironment (TME) to predict prognosis in MIBC and responses to adjuvant chemotherapy.

**Methods:**

We performed multiplex immunohistochemistry (IHC) profiling and quantification of immune and stromal cells (CD3, CD4, CD8, CD163, FoxP3, PD-1, and CD45, Vimentin, αSMA, PD-L1, Pan-Cytokeratin, Ki67) in 101 patients with MIBC receiving radical cystectomy. We used uni- and multivariate survival analyses to identify cell types predicting prognosis. Samples were subdivided using K-means clustering for Treg and macrophage infiltration resulting in 3 clusters, Cluster 1: Treg high, cluster 2: macrophage high, cluster 3: Treg and macrophage low. Routine CD68 and CD163 IHC were analyzed with QuPath in an extended cohort of 141 MIBC.

**Results:**

High concentrations of macrophages were associated with increased risk of death (HR 10.9, 95% CI 2.8–40.5; p < 0.001) and high concentrations of Tregs were associated with decreased risk of death (HR 0.1, 95% CI 0.01–0.7; p = 0.03) in the multivariate Cox-regression model adjusting for adjuvant chemotherapy, tumor and lymph node stage. Patients in the macrophage rich cluster (2) showed the worst OS with and without adjuvant chemotherapy. The Treg rich cluster (1) showed high levels of effector and proliferating immune cells and had the best survival. Cluster 1 and 2 both were rich in PD-1 and PD-L1 expression on tumor and immune cells.

**Conclusion:**

Treg and macrophage concentrations in MIBC are independent predictors of prognosis and are important players in the TME. Standard IHC with CD163 for macrophages is feasible to predict prognosis but validation to use immune-cell infiltration, especially to predict response to systemic therapies, is required.

**Supplementary Information:**

The online version contains supplementary material available at 10.1186/s12967-023-03949-3.

## Background

Bladder cancer (BCa) is causing about 213 000 deaths every year [1]. 25% of patients present with muscle-invasive bladder cancer (MIBC) at the time of diagnosis, which is associated with 5 year overall survival rates of about 50%. Radical cystectomy with lymphadenectomy is the standard therapy for patients with MIBC. Perioperative platin-based chemotherapies are recommended by guidelines to improve survival rates [2]. However, response rates to chemotherapies are limited and besides pathological staging and platin eligibility no markers exist to select patients for the application of perioperative chemotherapy [2]. Promising disease-free survival (DFS) rates for an adjuvant treatment with the immune checkpoint inhibitor Nivolumab led to its approval for patients with locally advanced and lymph node positive bladder cancer or patients not responding to neoadjuvant chemotherapy, when tumors show PD-L1 expression [3]. However, the application of adjuvant atezolizumab failed to show an improvement in DFS in a similar patient cohort [4]. In the latter study, high immune cell infiltration and basal-squamous features were associated with lower risk of recurrence [5]. Considering novel therapeutic approaches and the progress in precision medicine, further tumor classifications and markers besides clinical and pathological factors are necessary to select patients for either chemo- or immunotherapy in the adjuvant setting. The advances in immunomodulating therapies have increased the focus on the tumor microenvironment (TME) of MIBC, which can influence the response to therapy [5–12]. For example, the presence of tumor-infiltrating immune cells has been proposed as a positive prognostic factor and to be predictive for the response to perioperative chemotherapy as well as immunotherapy [8, 13, 14]. Moreover, tumor-associated macrophages (TAMs) are abundant in solid tumors and can promote tumor progression [15, 16]. In bladder cancer, high presence of TAMs is associated with poor prognosis [16–19].

Here, we investigated the presence and the impact of subsets of tumor-associated immune cells in MIBC on survival using multiplex immunohistochemistry.

### Cohort

Tissue/tumor samples and patient data used in this study were provided by the University Cancer Center Frankfurt (UCT). Written informed consent was obtained from all patients and the study was approved by the institutional review boards of the UCT and the ethical committee at the University Hospital Frankfurt (project-number: SUG-6-2018 and UCT-53-2021) which was conducted according to local and national regulations and to the Declaration of Helsinki.

A total of 145 formalin-fixed, paraffin-embedded (FFPE) tissue samples from patients with MIBC treated at the department of Urology, University Hospital Frankfurt from 2010 to 2020 were retrieved from the Dr. Senckenberg Biobank (SBB) at the Senckenberg Institute of Pathology. 

Histopathology of all cases was systematically re-reviewed by two genitourinary pathologists according to current WHO-criteria [20]. Histological subtypes were reported if at least 10% of tumor showed subtype histology including pure and mixed tumors.

### Immunohistochemical (IHC) analysis

The construction of the tissue microarray (TMA) has been described before [21]. In brief, tissue cores (1 mm) of representative tumor areas were transferred to a new block using the TMA Grandmaster (3DHISTECH, Budapest, Hungary). Hematoxylin and eosin stainings (H&E) were performed automatically (Sakura Finetek, Torrance, CA, USA) and IHC was performed using the DAKO Omnis staining system (Agilent, Santa Clara, CA, USA). We performed staining for Anti-CD68 (Clone KP1; DAKO, Agilent, Santa Clara, CA, ready to use) and CD163 (Clone MRQ-26; Cell Marque, Rockin, CA, USA, 1:100). TMA cores with absence of tumor, tissue or artefacts were excluded from the analysis. We used the open-source software QuPath (https://qupath.github.io) to quantify cells and DAB positive cells on TMA cores and calculate the percentage of positive cells [22]. The settings for the positive cell detection are displayed in Additional file 1: Figure S1.

The multiplex analysis was performed on 105 samples, which were stained with Opal 7‐Color Automation IHC Kits (Akoya Biosciences, Menlo Park, CA, USA) on a BOND‐RX Multiplex IHC Stainer (Leica, Wetzlar, Germany). We used the following primary antibodies on two panels: 1. T cell panel: anti‐CD163 (clone EPR19518; abcam, Cambridge, UK), anti‐CD4 (clone EPR6855; abcam, Cambridge, UK), anti‐CD3 (clone D7A6E; CellSignaling, Danvers, USA), anti‐PD-1 (polyclonal; Sigma, Darmstadt, Germany, HPA035981), anti‐CD8 (Clone C8/144B; DAKO, Agilent, Santa Clara, CA, USA), anti‐FoxP3 (clone 236A/E7; abcam, Cambridge, UK). 2. TME-panel: anti‐PD-L1 (clone SP142; abcam, Cambridge, UK), anti‐Pan-Cytokeratin (panCK) (clone C-11; abcam, Cambridge, UK) anti‐αSMA (clone 1A4; Sigma, Darmstadt, Germany), anti‐Ki67 (clone SP6; abcam, Cambridge, UK), anti-Vimentin (clone EPR3776; abcam, Cambridge, UK), and anti-CD45 (polyclonal; abcam, Cambridge, UK, ab10558). 4′,6‐diamidino‐2‐phenylindole (DAPI) (SouthernBiotech, Birmingham, AL, USA) was used for counterstaining of nuclei.

Corresponding secondary HRP-conjugated antibodies (Akoya Biosciences, Menlo Park, CA, USA, ARH1001A) and Opal fluorophores (Akoya Biosciences, Menlo Park, CA, USA, FP1500001KT, FP1487001KT, FP1488001KT, FP1495001KT, FP1497001KT, FP1501001KT) were used as described before [23, 24]. Images were acquired with the PhenoImager HT imaging system (Akoya Biosciences, Menlo Park, CA, USA) and analyzed using the Phenotyping application of the inForm software V2.5 (Akoya Biosciences, Menlo Park, CA, USA). Briefly, after tissue (stroma/tumor) and cell segmentation, populations were identified with markers by using the phenotyping algorithm provided in the in Form software. The following cells were identified: double negative (DN) T-cells: CD3+ CD4-CD8-; T-helper cells: CD3+ CD4+ CD8-FoxP3-; cytotoxic T cells: CD3 + CD8+ CD4-; PD-1 positive cytotoxic T cells: CD3 + CD8 + PD-1 + ; Macrophages: CD163 + ; regulatory T cells (Tregs): CD3 + CD4 + FoxP3 + ; fibroblasts: αSMA + Vimentin + CD45-; CD45 + immune cells: CD45 + PD-L1-panCK-Vimentin-; tumor cells: panCK + CD45-Vimentin-; PD-L1 positive tumor cells: panCK + PD-L1 + Vimentin-CD45-; PD-L1 positive immune cells: CD45+ PD-L1+ panCK-Vimentin- proliferating immune cells: CD45+ Ki67+ ; proliferating tumor cells: panCK + Ki67 + Vimentin-CD45-. Since the vast majority of immune cells was detected in the assigned stroma area, we used cell counts of the whole tissue area to avoid incorrect tissue segmentation bias (Additional file 1: Figure S2). TMA cores with either absence of representative tumor tissue or presence of staining artifacts were excluded from the analysis.

### Statistical analysis

We performed descriptive statistics of all data. Subgroup comparisons were tested with Mann-Whitney *U* test and Chi^2^ test for nonparametric variables, two-sided t-test and ANOVA for parametric variables.

For the visualization, the multiplex IHC data were standardized using JMP (SAS Institute Inc.) by the conversion of the data to a mean of 0 and a standard deviation (SD) of 1 (z-score: *(z* = *xi − mean(x)/SD(x))*.

For the survival analysis, only patients with radical cystectomy in “curative intent” were included: patients with metastatic disease or that received neoadjuvant chemotherapy were excluded from the analyses. We defined the overall survival (OS) as main endpoint of interest, which was defined as time interval between surgery and death.

We used the Kaplan-Meier method to estimate and illustrate survival probabilities as well as uni- and multivariate Cox’s proportional models to estimate the hazard ratio (HR) and the corresponding 95% confidence intervals. A significance level of α = 5% was used. Statistical analyses were performed using JMP (SAS Institute Inc.) Version 16.2.0 and R Studio (Version 2022.02.3). We used the cut-off finder to calculate the cut-off for the percentage of CD68 and CD163 positive cells with the minimum log-rank P-value method [25].

## Results

### Patient characteristics

Of the 105 samples stained with multiplex immunohistochemistry, four samples analyzed with the T cell panel and five samples analyzed with the TME panel were excluded due to artefacts or tissue detachment. 101 patients with MIBC were available for pathological evaluation and final statistical analysis. Clinico-pathological details of the cohort are summarized in Table 1.

**Table 1:** Clinico-pathological details of 101 patients receiving radical cystectomy evaluable on TMA for T cell and TME-panel with multiplex immunohistochemistry.

		n = 101
Median age (IQR)		68 (59–75)
Gender		
Male		78 (77%)
Female		23 (23%)
Max. tumor-stage		
pT2		24 (24%)
pT3		57 (56%)
pT4		20 (20%)
Lymph node status		
pN0		49 (49%)
pN+/pNx		52 (51%)
Histological subtype		
NOS		73 (72%)
Squamous		11 (11%)
Micropapillary		6 (6%)
Neuroendocine		3 (3%)
Sarcomatoid		2 (2%)
Plasmacytoid		2 (2%)
Other (2 Lymphoepithelial, 1 Glandular, 1 Giant cell)		4 (4%)
Min. 2 cycles adjuvant chemotherapy		
No		67 (66%)
Yes		34 (34%)

*IQR* interquartile range, *NOS* not otherwise specified

### Survival analysis

We assessed survival rates of 101 patients with adequate follow up that received radical cystectomy. Median follow-up was 66 months (IQR 31–97 months). Thirty-four patients received at least two cycles of adjuvant chemotherapy.

Known predictive factors such as tumor and lymph node stage, as well as the application of adjuvant chemotherapy, were significantly associated with OS (Table 2, Additional file 1: Figures S4–S5). Patients receiving adjuvant chemotherapy had median OS of 32 months (95% CI 20-not reached) and patients without adjuvant chemotherapy had median OS of 11 months (95% CI 6–21), *p*(log-rank) = 0.005 (Additional file 1: Figure S3).

**Table 2:** Cox-Regression model for overall survival (OS).

		Hazard ratio (univariate)	*p-value*	Hazard ratio (multivariate)	*p-value*
Gender	Female vs. male	1.3 (0.7–2.3)	*0.43*		
Tumor stage	pT3 vs. pT2	2.2 (1.1–4.5)	**0.02**	2.47 (1.2–5-1)	**0.01**
	pT4 vs. pT2	4.1 (1.8–9.0)	**< 0.001**	4.1 (1.8–9.5)	< **0.001**
Lymph node status	pN+ /pNx vs. pN0	2.1 (1.3–3.5)	**0.004**	2.5 (1.4–4.2)	**0.001**
Adjuvant chemotherapy	yes vs. no	0.5 (0.3–0.8)	**0.007**	0.3 (0.2–0.5)	< **0.001**
CD3+ T cells	continuous	0.3 (0.04–1.6)	*0.20*		
CD4+ T cells	continuous	0.07 (0.0–1.6)	*0.25*		
CD8+ T cells	continuous	0.4 (0.04–2.2)	*0.38*		
Tregs	continuous	0.2 (0.03–0.8)	**0.04**	0.1 (0.0–0.7)	**0.04**
CD45+ immune cells	continuous	0.6 (0.2–1.6)	*0.3*		
Fibroblasts	continuous	2.0 (0.6–5.9)	*0.2*		
PD-L1+ immune cells	continuous	0.7 (0.17–2.4)	*0.61*		
Proliferating tumor cells	continuous	0.6 (0.02–5.4)	*0.76*		
Proliferating immune cells	continuous	0.2 (0.04–0.82)	**0.04**	0.2 (0.03–0.7)	**0.02**
Macrophages	continuous	4.0 (1.25–11.52)	**0.01**	10.9 (2.8–40.5)	< **0.001**
K-means clusters for Tregs and macrophages	2 vs. 1	5.4 (1.54–18.8)	**0.008**	3.5 (1.0–12.5)	**0.05**
	2 vs. 3	2.1 (1.15–3.38)	**0.02**	2.4 (1.3–4.4)	**0.006**
	3 vs. 1	2.6 (0.8–8.3)	*0.1*	1.5 (0.4–4.8)	*0.5*

Variables with significant prediction on OS were added to the multivariate model adjusting for tumor, lymph node status and adjuvant chemotherapy. K-means clusters for macrophages and Tregs was performed using their ratio to all counted cells. Cluster 1: Treg high; Cluster 2: macrophage high; Cluster 3: Treg low and macrophage low

In the univariate survival analyses, we further identified patients with a high concentration (cells/mm^2^) of Tregs to have a decreased risk of death (HR 0.17; 95% CI 0.03–0.76; *p* = 0.04), and patients with a high concentration of macrophages to have an increased risk of death (HR 4.0; 95% CI 1.25–11.52; *p* = 0.01). The high presence of Tregs, macrophages, and proliferating immune cells (CD45 + Ki67 +) each was confirmed as an independent prognostic factor in the multivariate cox regression model combining variables with significant influence on OS (adjuvant chemotherapy, tumor, and lymph node stage).

**Fig. 1:**
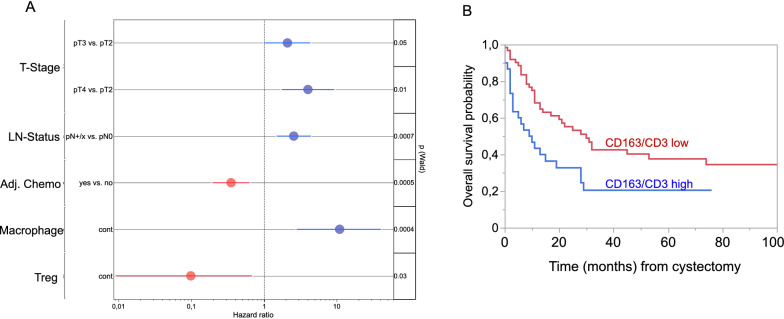
**A**: Forrest plot for the multivariate survival analysis including macrophages, Tregs, tumor stage (T-Stage), lymph node status (LN-Status) and adjuvant chemotherapy (Adj. Chemo). **B**: Overall survival probability for the ratio of macrophages to CD3 + T-cells p(Log-Rank) = 0.008. Patients with a ratio of ratio of < 50 macrophages to CD3+ T-cells were stratified as low and patients with a ratio of ≥ 50 as high. The cut-off was calculated using the cut-off finder with the minimum log-rank P-value method

Findings were also confirmed by calculating the ratio of macrophages to CD3+ T cells. A cut-off was defined using the minimum log-rank p-value method [24]. Patients with a ratio of < 50 had median OS of 30 months (95% CI 14–74) and patients with a ratio of ≥ 50 had median OS of 9.5 months (95% CI 3–19), *p* (log-rank) = 0.008 (Fig. 1).

### Clustering of tumors according to macrophage and Treg infiltration

**Fig. 2:**
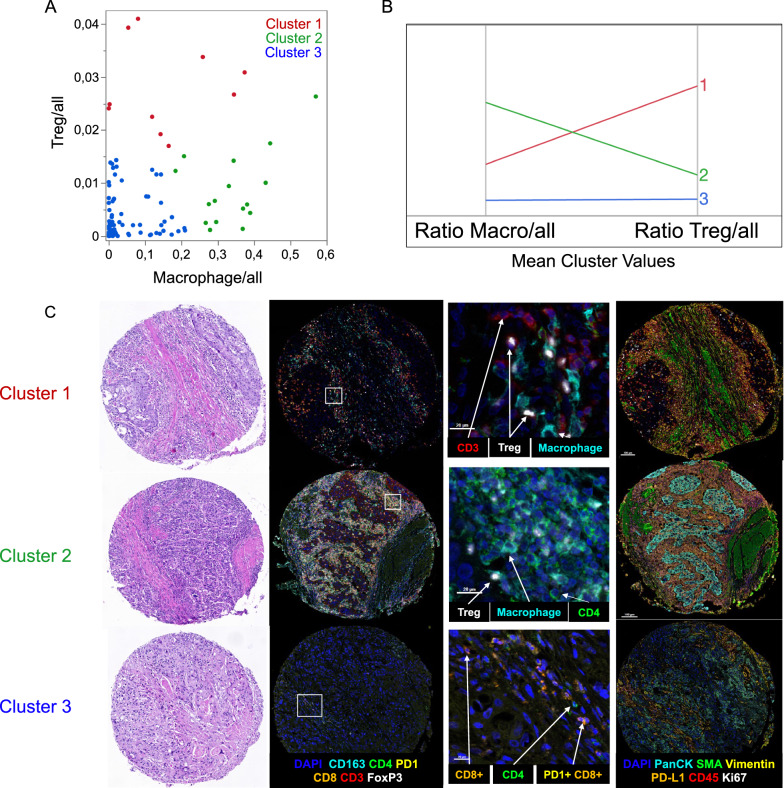
**A**: The distribution plot for the ratio of macrophages and Tregs shows individual samples colored according to the assigned K-means cluster. **B**: Parallel coordinate diagram of mean cluster values for macrophages and Tregs. Cluster 1 (red) is characterized by high Treg infiltration, cluster 2 (green) is characterized by high macrophage infiltration, cluster 3 (blue) has low Treg and low macrophage infiltration. **C**: Each line shows a representative case of the three clusters with HE-staining (magnification 7.2x) and multiplex immunohistochemistry for T-cell and TME markers: Nuclei were counterstained with DAPI (blue). T-cell panel: CD163 (cyan), CD4 (green), PD-1 (yellow), CD8 (orange), CD3 (red), FoxP3 (white); (B) TME panel: PanCK (cyan), αSMA (green), Vimentin (yellow), CD45 (orange), PD-L1 (red), Ki67 (white)

We next performed K-means clustering of samples for macrophages and Tregs using their ratio to all counted cells on a TMA spot. Samples were clustered into three groups. Cluster 1 with 10 patients was driven by patients with high Treg concentration. Sixteen patients were assigned into cluster 2, showing high macrophage concentration and lower Treg concentration compared to cluster 1. Patients in cluster 3 had low Treg and low macrophage infiltration (Fig. 2).

**Fig. 3:**
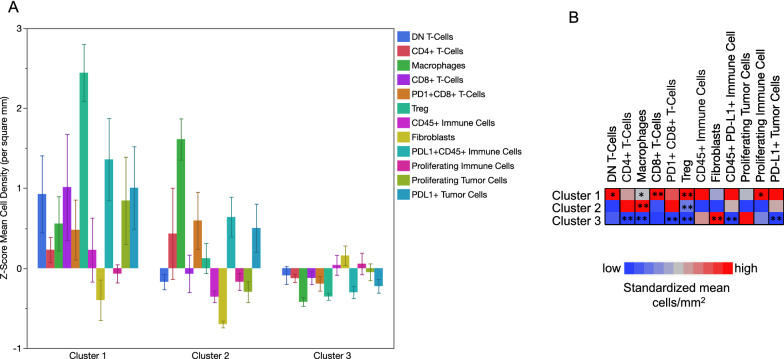
**A**: Z-Scores of cells/mm^2^ for each cell type represented as bars in the three clusters. Error bars show standard errors of means. **B**: Heatmap of Z-Scores showing the mean cell density score for the clusters in rows. P values were calculated using the Wilcoxon rank sum test. * p < 0.05; ** p < 0.01

Besides Treg and macrophage infiltration, the clusters showed different characteristics in the TME (Fig. 3, Additional file 1: Figure S6–S7). Cluster 1 showed higher concentration of other immune cells and proliferating immune cells (CD45 + Ki67 +) compared to cluster 2 and 3 (*p* = 0.04 and *p* = 0.05, Mann-Whitney *U* test). A positive correlation between Tregs and proliferating immune cells was observed (*r* = 0.47*; p* < 0.001; Additional file 1: Figure S8). Concentrations of proliferating tumor cells (panCK + Ki67 +) did not significantly differ between the three clusters. Cluster 3 was enriched in fibroblasts. Further significant correlations between the clusters are included in Table 3. The clinical and pathological characteristics (i.e. tumor and lymph node stage, age, gender, histological subtype and application of adjuvant chemotherapy) did not differ significantly between the three clusters (Additional file 1: Table S1). However, we noticed that histological subtypes such as micropapillary, neuroendocrine, plasmacytoid or sarcomatoid tumors were enriched in the immune depleted cluster 3.

**Table 3:** Association detected cells per square mm within the calculated K-means clusters according to concentration of Tregs (FoxP3 +) and macrophages (CD163 +). Level of significance (p-value) was calculated using Kruskal-Wallis test

		All patient n = 101	Cluster 1 treg high (n = 10)	Cluster 2 macrophage high (n = 16)	Cluster 3 immune low (n = 75)	*p*-value
Mean cell density/mm^2^, (95% CI)	Double negative CD3 + T cells	13.4 (9.1–17.8)	33.8 (10.0–57.7)	9.7 (5.2–14.2)	11.5 (6.7–16.3)	0.04
	CD4+ T cells	65.6 (25.7–105.5)	112.3 (39.9–184.7)	153.3 (0–398.8)	40.7 (19.6–61.8)	0.007
	Macrophages	296.2 (217.3–375.1)	519.1 (211.8–826.4)	940.7 (721.1–1160.2)	129.0 (86.4–171.5)	< 0.001
	CD8+ T cells	52.5 (33.6–71.4)	149.6 (92.5–206.6)	46.0 (1.0–91.0)	41.0 (20.1–61.8)	0.01
	PD1 + CD8 + T cells	37.8 (19.4–56.1)	82.4 (26.8–138.0)	93.1 (49.2–137.1)	20.0 (0–40.3)	< 0.001
	Tregs	21.7 (15.1–28.3)	103.1 (76.2–130.0)	25.9 (12.5–39.3)	10.0 (7.1–12.9)	< 0.001
	CD45 + PD-L1- immune cells	351.1 (267.6–434.6)	448.6 (72.9–824.2)	205.2 (140.4–296.9)	369.48 (266.7–472.2)	0.8
	Proliferating immune cells	46.9 (35.6–58.1)	94.6 (25.0–164.3)	30.1 (14.3–45.8)	44.0 (32.1–55.9)	0.08
	Proliferating tumor cells	102.9 (63.0–142.7)	88.0 (37.6–138.3)	68.1 (23.7–112.5)	112.4 (59.5–165.3)	0.5
	Fibroblasts	135.5 (105.8–165.2)	80.6 (0–167.2)	35.4 (21.6–49.2)	164.5 (127.9–201.1)	< 0.001
	PD-L1 + tumor cells	41.5 (22.5–60.5)	134.8 (24.6–244.9)	87.4 (26.7–148.0)	19.0 (3.0–34.9)	< 0.001
	CD45 + PD-L1 + immune cells	807 (171–1090)	2038 (619–3126)	1377 (711–2074)	511 (132–701)	< 0.001

**Fig. 4:**
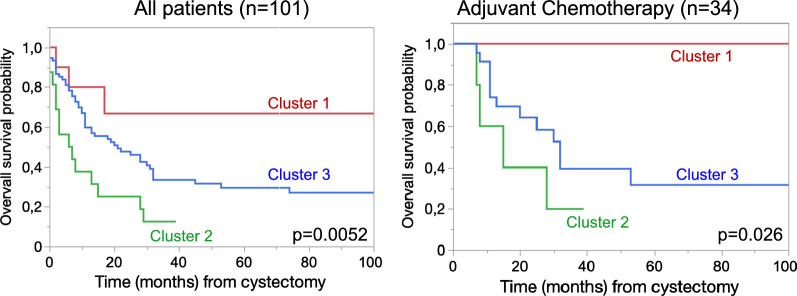
Kaplan–Meier curves for overall survival probability for all patients and patients receiving at least two cycles of adjuvant chemotherapy. Survival was significantly worse in cluster 2 (high macrophage infiltration) compared to cluster 1 or 3

We assessed survival rates of patients stratified into three clusters for macrophage and Treg infiltration. Patients in cluster 2 with high concentration of macrophages had the poorest OS with median OS probability of 11 months (95% CI 6.2–19.3). Patients in cluster 1 had the best survival with median OS probability of 82 months (95% CI 26.6–255). Patients in the immune cell depleted cluster 3 showed an intermediate OS-rate with median OS probability of 35 months (95% CI 26.0–46.9). The differences in OS were also present when only including patients receiving adjuvant chemotherapy after radical cystectomy (*p* = 0.026), (Fig. 4). The influence on survival was confirmed by combining the clusters with other prognostic factor (adjuvant chemotherapy, tumor and lymph node stage) in the multivariate cox regression model (Table 2) for cluster 2 vs. 1 with a HR of 3.5 (95% CI 1.01–12.5, *p* = 0.05) and for cluster 2 vs. 3 with a HR of 2.4 (95% CI 1.3–4.4; *p* = 0.006). The OS of patients in cluster 1 did not differ significantly compared to patients in cluster 3 (HR 1.5; 95% CI 0.4–4.8), *p* = 0.5).

### Validation using standardized IHC

**Fig. 5:**
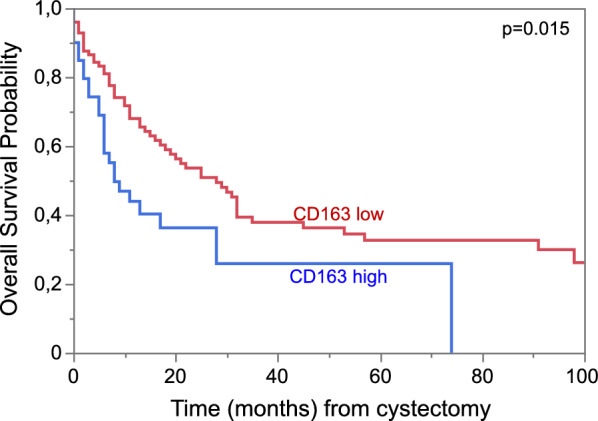
Kaplan–Meier curves for overall survival probability for patients in the extended cohort (n = 139) stratified for low and high infiltration of (TAMs) CD163+ cells measured with IHC and analyzed with QuPath algorithm with a cut-off of 20%/all cells; p(Log-Rank) = 0.02

To confirm our findings and facilitate the use of macrophage infiltration as a prognostic marker, we performed routinely used IHC of CD163 and CD68 on an extended cohort of 145 patients with MIBC, including the 101 patients used above, treated with radical cystectomy. 139 and 141 patients were evaluable for CD163 and CD68, respectively. Quantification of positive cells was performed with QuPath and cut-offs of 5% CD68 and 20% CD163 positive cells were calculated to stratify patients with low and high macrophage infiltration [22, 25]. Correlation between CD68 and CD163 expression was high in routine IHC (R^2^ = 0.61; p < 0.001) but lower when compared to CD163 expression used in the multiplex IHC-panel (R^2^ = 0.38 for CD163 and R^2^ = 0.17 for CD68 p < 0.001; Additional file 1: Figure S10). High levels of the marker CD68 showed a trend towards decreased survival, but missed significance to predict OS (HR 1.5, 95% CI 0.97–2.4; p = 0.06; Additional file 1: Figure S9). Patients with high CD163 expression had increased risk of death (HR 1.8, 95% CI 1.1–2.9; p = 0.02) (Fig. 5). Clinical and pathological characteristics of patients in each group are specified in the Additional file 1 (Additional file 1: Table S2).

## Discussion

With the introduction of immune-checkpoint inhibitors as therapeutic option in muscle invasive and metastatic urothelial carcinoma, efforts have been made to improve management of patients [3, 4, 26–29]. However, only a subset of patients responds to immunotherapies and/or are resistant to chemotherapies. Hopes were put into molecular subtypes to stratify patients, that respond from systemic therapies, but contradictory results and the diversity of molecular subtype taxonomy have hindered the use and clinical translation so far [8, 30–34]. Molecular subtypes lack prospective validation and recent publications propose that high immune infiltration increases sensitivity to chemotherapy and might serve as predictive marker [6–9].

By using an unbiased automated approach with 7‐color multiplex IHC to detect cells we were able to quantify different cell types and their association within a tumor. Our results clearly point out that high levels of TAMs are a negative prognostic marker in patients with MIBC undergoing radical cystectomy with and without adjuvant chemotherapy. Tumors with high macrophage and low Treg infiltration had less CD8 + T cells, but where rich in PD-1/PD-L1 expression of tumor and immune cells. This combination could cause poor survival and poor response to chemotherapy. On the other hand, Tregs, identified by FoxP3, were associated with high presence of CD8 + T cells and proliferating immune cells that have a positive effect on survival. Most patients were stratified into cluster 3, showing overall low immune cell infiltrates, having inferior OS than patients in the Treg rich cluster, but still superior to the macrophage rich cluster.

Higher stromal immune cell infiltration in MIBC has been shown to be associated with increased anti-tumor immune response, response to chemotherapy, and improved survival rates [6–8, 12, 13]. While this effect is mostly attributed to cytotoxic T-cells [7, 10, 11, 13, 18], anti‐inflammatory macrophages are considered to be tumor‐promoting and have been described as a negative prognostic predictor for multiple cancer entities [15, 23, 35–37]. In bladder cancer several studies have shown TAMs to be associated with poor clinical outcome [15, 18, 38, 39]. Recently it was shown that TAMs expressing IL10 are associated with inferior prognosis of MIBC, but these patients showed a superior response to chemotherapy [37]. In another cohort, patients with the presence of Galectin-9 expressing TAMs had decreased OS and RFS, but an improved response to adjuvant chemotherapy [39]. In our hands, patients in cluster 2, which is defined by high presence of macrophages, had poor outcome also when receiving adjuvant chemotherapy. It would be relevant to explore this feature in the setting of neoadjuvant chemotherapy and adjuvant immunotherapy. Furthermore, preclinical bladder cancer models suggest inhibition of macrophage recruitment via blockage of CCL2 can lead to a reduction of lymph node metastases and increased survival after chemotherapy [40, 41]. For other tumor entities it was shown that TAM depletion by blocking colony-stimulating factor 1 receptor (CSF1R) promoted an anti-tumor immune response and lead to an enhanced response to anti PD-L1 treatment [42–44].

Concerning the presence of regulatory T cells (Tregs), ambiguous results have been published. Horn et al. did not observe a correlation when using FoxP3 as a single marker for Tregs, but described a slightly shorter OS in patients with an increased FoxP3/CD3-ratio [45]. In contrast, a small study by Winerdal et al. found higher infiltration of FoxP3 + T cells to be correlated with better survival [46]. Although Tregs seem to have negative impact on patient survival across a number of tumor entities, this prognostic effect varies between different cancer types [47]. In the present study, tumors in the Treg rich cluster were enriched with other immune cells like CD3+ , CD8+ , proliferating immune cells and PD-L1 expressing immune and tumor cells. Correlations of Treg and T cell scores have also described by others [15]. Although FoxP3 is often considered a specific marker for Tregs, it has also been described as a marker of activated T cells without regulatory or immunosuppressive functions [48–50]. Thus, FoxP3 positive cells might function as representatives of high immune-cell infiltration, which overall leads to favorable survival. A multiomic study by Taber et al. showed similar results regarding the spatial immune cell analysis claiming that higher PD-L1 expression leads to better response rates of neoadjuvant chemotherapy in MIBC, and immune infiltrated and excluded tumors have better treatment outcome compared to immune desert tumors [8]. High expression of PD-1/PD-L1 can represent T cell dysfunction and is often correlated to poor prognosis in urothelial cancer [51–53]. However, a lower risk of recurrence and better prognosis for patients with high PD-1 and PD-L1 has also been described [54, 55]. Since a significant number of immune cells with PD-L1 expression was observed in cluster 1 as well as cluster 2, the underlying biology of cells expressing immune checkpoint molecules and how this influences survival needs to be further investigated in detail.

Other studies focused either on stromal or tumor infiltrating immune cells or separated tumors into immune excluded, infiltrated or immune desert [8, 10, 18, 38]. We also separately analyzed Tregs and macrophages in tumor- and stroma-areas. Both cell types were predominantly located in the stroma and had prognostic influence when being present in the stroma, but not in the tumor (see Additional file 1: Table S3 and Additional file 1: Figure S2). However, the predictive value was higher when analyzing the whole tissue area. Furthermore, a non‐biased approach of a representative TMA core or tumor area could be more reproducible.

The application of multiplex IHC and multispectral imaging is a powerful tool to image and characterize tumors and the cellular subsets. However, it is not applicable in clinical routine and the reproducibility is dependent on staining, scanning and the cell detection algorithm. Using routine IHC-staining and digital analysis with QuPath of CD163 in an extended cohort confirmed the prognostic value of the marker. This result had been confirmed by other groups before [15, 19, 56]. In a study by Taubert et al., CD68 was also proposed to be predictive to stratify patients, which is in line with our results, even though CD68 as a macrophage marker did not reach significance in our cohort (HR 1.5, 95% CI 0.97–2.4; p = 0.06) [19]. This might be an issue of sample size. CD163 is a pan‐macrophage marker, which can be induced under M2 conditions, but is still detectable at protein level in other macrophages [23, 57, 58]. It has been described as a marker of alternatively activated and therefore, presumably, tumor‐promoting macrophages [35, 36]. Although the correlation between CD68 and CD163 was high, some cells expressed CD163 without CD68. Adding further markers of macrophage subsets might be instrumental to better characterize, e.g., M1/M2 polarization and is planned for future experiments. Moreover, further research will focus on a more detailed analysis of macrophage subsets, such as resident versus recruited macrophages or subsets expressing markers such as CD206, and their functions [23, 59].

The application of two multiplex IHC panels followed by the algorithm-based cell analysis enabling a morphological analysis with single cell resolution together with a well characterized MIBC cohort are strengths of this study. The data allow detailed analyses of the TME to confirm TAMs as an important predictor of poor survival. However, our study has important limitations. The retrospective design and limited number of patients receiving chemotherapy limits the predictive value. Using TMAs might not reflect bladder cancer heterogeneity sufficiently. Furthermore, only one marker (CD163) was used to identify macrophages. The antibodies used for the multiplex IHC were not the same as used in pathological routine. This can explain differences between the multiplex IHC cohort and the confirmation cohort. Especially regarding the PD-L1 expression, different antibodies can cause variable results and are not transferable from multiplex IHC to clinical routine [60]. Finally, the mode of action of the Treg rich cluster and PD-1/PD-L1 expressing cells remains to be revealed.

## Conclusion

Our data show that high infiltration of TAMs is associated with poor survival with and without adjuvant chemotherapy in MIBC. A more detailed characterization of TAMs will uncover subsets of macrophages that are prognostic and could be potential targets in bladder cancer therapy. Besides macrophages, high levels of Tregs were correlated with other proliferating immune cells, which was correlated with improved survival rates, indicating that cells in the tumor microenvironment have more impact on patient survival than tumor intrinsic subtypes. This needs to be translated into predictive use to guide therapeutic decisions, which requires prospective validation with standardized measurements and unbiased cell counts and calculations.

## Supplementary Information

**Additional file 1:** **Table S1.** Association of clusters with clinical and pathological patient characteristics. Level of significance (p-value) was calculated using Chi2-test or ANOVA, respectively. IQR=interquartile range; NOS=not otherwise specified. **Table S2.** Clinical and pathological patient characteristics did not differ significantly between low and high CD163+ macrophage infiltration. **Table S3.** Cox-Regression model for overall survival (OS) with immune cells counted only in the stroma. The stroma the stroma area was assigned using the algorithmic tissue separation of the inForm® Tissue Analysis Software. Variables with significant prediction on OS were added to the multivariate model adjusting for tumor, lymph node status and adjuvant chemotherapy. K-means clusters for macrophages and Tregs was performed using cells per square mm counted in the stroma. Cluster 1: Treg high; Cluster 2: macrophage high; Cluster 3: Treg low and macrophage low. **Figure S1.** QuPath Settings for positive cell detection for CD163 (upper image) and CD68 (lower image). Magnification 400x. **Figure S2.** Number of cells (per mm2) detected in the stroma and tumor area using the algorithmic tissue separation of the inForm® Tissue Analysis Software. **Figure S3.** Kaplan–Meier curves for overall survival probability for patients with (n=34) and without adjuvant chemotherapy (n=67); p(log-rank) = 0.005. **Figure S4.** Kaplan–Meier curves for overall survival probability stratified for patients by pathological tumor stage; p(log-rank) = 0.001. **Figure S5.** Kaplan–Meier curves for overall survival probability stratified for patients by pathological lymph node stage; p(log-rank) = 0.0002. **Figure S6.** Cell types (mean cell density/mm2) represented in the three clusters. Error bars show standard errors of means. **Figure S7.** Heatmap of Z-Scores (z = xi −mean(x)/ st.dev(x)) for each cell type and each sample. **Figure S8.** Correlation plot of the cell density of Treg (FoxP3+) vs. proliferating immune cells (CD45+Ki67+). **Figure S9.** Kaplan–Meier curves for overall survival probability stratified for patients by the macrophage marker CD68 with a cut-off 5%/all cells; p(log-rank) = 0.055. **Figure S10.** Correlation plot of routine IHC for CD163 and CD68 vs. CD163 in the multiplex IHC panel (A). And Correlation between CD68 and CD163 expression in routine IHC (B).

## Data Availability

Data that support the findings of this study are available from the corresponding author upon reasonable request.
