# Supplementary material for: Tumor-associated macrophages and Tregs influence and represent immune cell infiltration of muscle-invasive bladder cancer and predict prognosis
Source: J Transl Med. 2023 Feb 15;21:124. doi: 10.1186/s12967-023-03949-3 (PMC9930232; doi:10.1186/s12967-023-03949-3)
Supplement: Supplementary file 1 — Additional file 1: Table S1. Association of clusters with clinical and pathological patient characteristics. Level of significance (p-value) was calculated using Chi2-test or ANOVA, respectively. IQR=interquartile range; NOS=not otherwise specified. Table S2. Clinical and pathological patient characteristics did not differ significantly between low and high CD163+ macrophage infiltration. Table S3. Cox-Regression model for overall survival (OS) with immune cells counted only in the stroma. The stroma the stroma area was assigned using the algorithmic tissue separation of the inForm® Tissue Analysis Software. Variables with significant prediction on OS were added to the multivariate model adjusting for tumor, lymph node status and adjuvant chemotherapy. K-means clusters for macrophages and Tregs was performed using cells per square mm counted in the stroma. Cluster 1: Treg high; Cluster 2: macrophage high; Cluster 3: Treg low and macrophage low. Figure S1. QuPath Settings for positive cell detection for CD163 (upper image) and CD68 (lower image). Magnification 400x. Figure S2. Number of cells (per mm2) detected in the stroma and tumor area using the algorithmic tissue separation of the inForm® Tissue Analysis Software. Figure S3. Kaplan–Meier curves for overall survival probability for patients with (n=34) and without adjuvant chemotherapy (n=67); p(log-rank) = 0.005. Figure S4. Kaplan–Meier curves for overall survival probability stratified for patients by pathological tumor stage; p(log-rank) = 0.001. Figure S5. Kaplan–Meier curves for overall survival probability stratified for patients by pathological lymph node stage; p(log-rank) = 0.0002. Figure S6. Cell types (mean cell density/mm2) represented in the three clusters. Error bars show standard errors of means. Figure S7. Heatmap of Z-Scores (z = xi −mean(x)/ st.dev(x)) for each cell type and each sample. Figure S8. Correlation plot of the cell density of Treg (FoxP3+) vs. proliferating immune cells (CD45+Ki67+). [file 12967_2023_3949_MOESM1_ESM.pdf]

Additional file

Tumor-associated macrophages and Tregs influence and represent immune cell infiltration of muscle-invasive bladder cancer and predict prognosis

Table S1

Variable		All patients n=101	Cluster 1 Treg high (n=10)	Cluster 2 Macrophage high (n=16)	Cluster 3 Immune low (n=75)	*p*
**Median Age (IQR)**		68 (59-75)	69.5 (52-80)	71 (58-72)	68 (59-75)	0.9
**Gender**	Male	78 (77%)	7 (70%)	12 (75%)	59 (79%)	0.8
	Female	23 (23%)	3 (30%)	4 (25%)	16 (21%)	
**Max. tumor-stage**	pT2	24 (24%)	3 (30%)	3 (19%)	18 (24%)	0.9
	pT3	57 (56%)	6 (60%)	9 (56%)	42 (56%)	
	pT4	20 (20%)	1 (10%)	4 (25%)	15 (20%)	
**pN Stage**	pN0	49 (49%)	5 (50%)	7 (44%)	37 (49%)	0.9
	pN1/pNx	52 (51%)	5 (50%)	9 (56%)	38 (51%)	
**Histological subtype**	NOS	73 (72%)	6 (60%)	13 (81%)	54 (72%)	0.4
	Squamous	11 (11%)	2 (20%)	1 (6%)	8 (11%)	
	Micropapillary	6 (6%)	0	1 (6%)	5 (7%)	
	Neuroendocine	3 (3%)	0	0	3 (4%)	
	Sarcomatoid	2 (2%)	0	0	2 (3%)	
	Plasmacytoid	2 (2%)	0	0	2 (3%)	
	Other (2 Lymphoepitheli al, 1 Glandular, 1 Giant cell)	4 (4%)	2 (20%)	1 (6%)	1 (1%)	
**Adjuvant chemotherapy**	No	67 (66%)	4 (40%)	11 (69%)	52 (69%)	0.2
	Yes	34 (34%)	6 (60%)	5 (31%)	23 (31%)	

Table S1: *Association of clusters with clinical and pathological patient characteristics. Level of significance (p-value) was calculated using Chi^2-test or ANOVA, respectively. IQR= interquartile range; NOS=not otherwise specified*

Table S2

		CD163 low (n=99)	CD163 high (n=40)	***p***
**Gender**	Female	19 (19%)	10 (25%)	0.5
	Male	80 (81%)	30 (75%)	
**Age**	Median (IQR)	68 (60-76)	71 (59-78)	0.2
**Tumor stage**	pT2	32 (32%)	7 (18%)	0.2
	pT3	51 (52%)	23 (58%)	
	pT4	16 (16%)	10 (25%)	
**Lymph node stage**	pN0	50 (51%)	17 (43%)	0.4
	pN+/pNx	49 (49%)	23 (57%)	
**Adjuvant Chemotherapy**	no	70 (71%)	30 (75%)	0.6
	yes	29 (29%)	10 (25%)	

Table S2: *Clinical and pathological patient characteristics did not differ significantly between low and high CD163+ macrophage infiltration.*

Table S3

		Hazard ratio univariate	p	Hazard ratio multivariate	p
**CD3 T-cells (Stroma)**	continuous	0.3 (0.06-1.3)	0.1		
**CD4 T-cells (Stroma)**	continuous	0.2 (0.0-1.9)	0.3		
**CD8 T-cells (Stroma)**	continuous	0.7 (0.1-3.2)	0.7		
**Tregs (Stroma)**	continuous	0.2 (0.03-0.8)	0.05	0.2 (0.0-1.3)	0.1
**Macrophages (Stroma)**	continuous	3.6 (1.1-10.6)	0.03	6.3 (1.8-21.0)	0.004
**K-Means Clusters for Tregs + Macrophages in Stroma**	2 vs. 1	4.6 (1.3-16.1)	0.02	3.1 (0.8-10.9)	0.08
	2 vs. 3	2.0 (1.1-3.6)	0.02	2.2 (1.2-4.1)	0.01
	3 vs. 1	2.3 (0.7-7.4)	0.2	1.4 (0.4-4.7)	0.6

Table S3: *Cox-Regression model for overall survival (OS) with immune cells counted only in the stroma. The stroma the stroma area was assigned using the algorithmic tissue separation of the inForm® Tissue Analysis Software. Variables with significant prediction on OS were added to the multivariate model adjusting for tumor, lymph node status and adjuvant chemotherapy. K-means clusters for macrophages and Tregs was performed using cells per square mm counted in the stroma. Cluster 1: Treg high; Cluster 2: macrophage high; Cluster 3: Treg low and macrophage low*

Figure S1

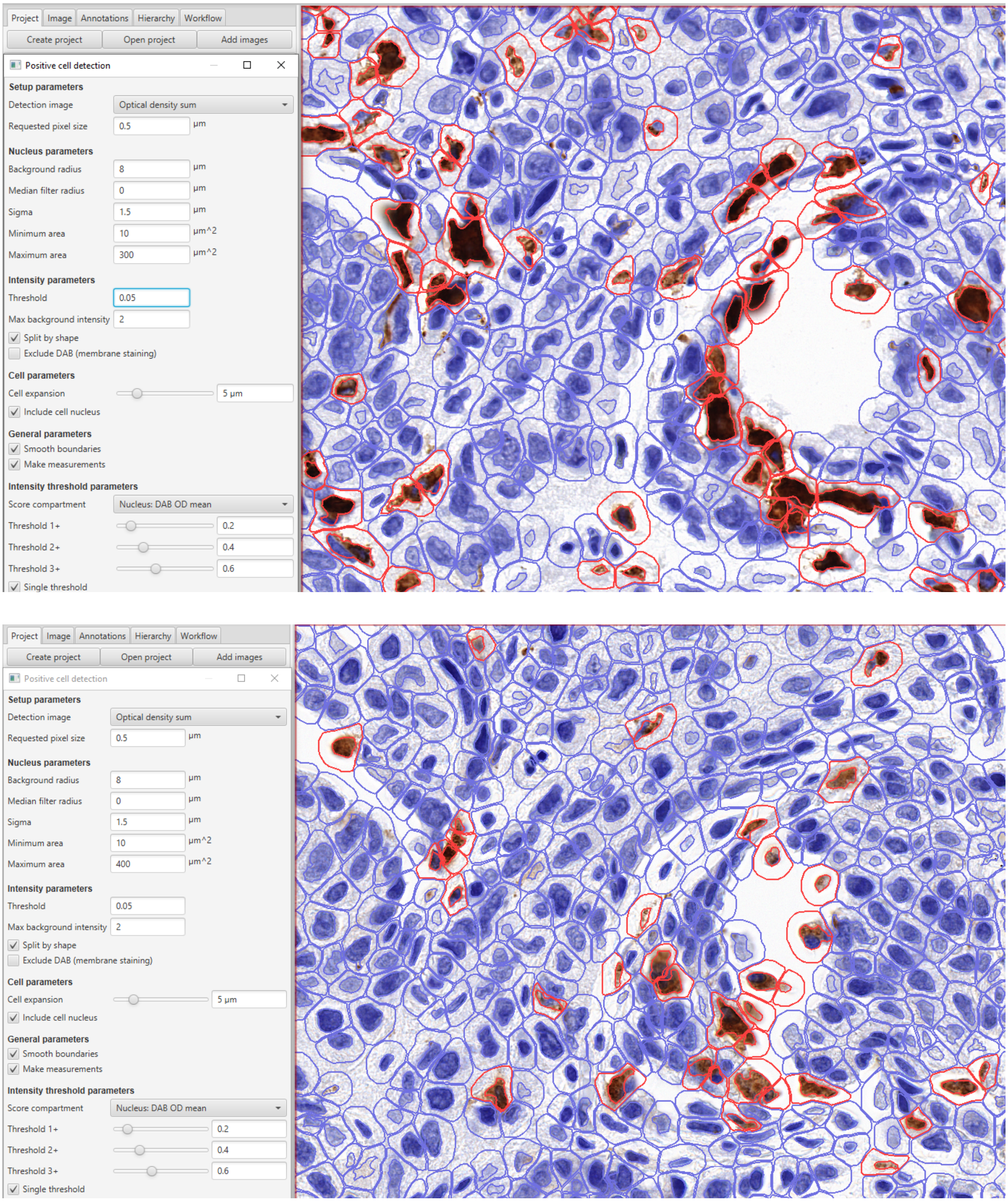

Figure S1: *QuPath Settings for positive cell detection for CD163 (upper image) and CD68 (lower image). Magnification 400x.*

Figure S2

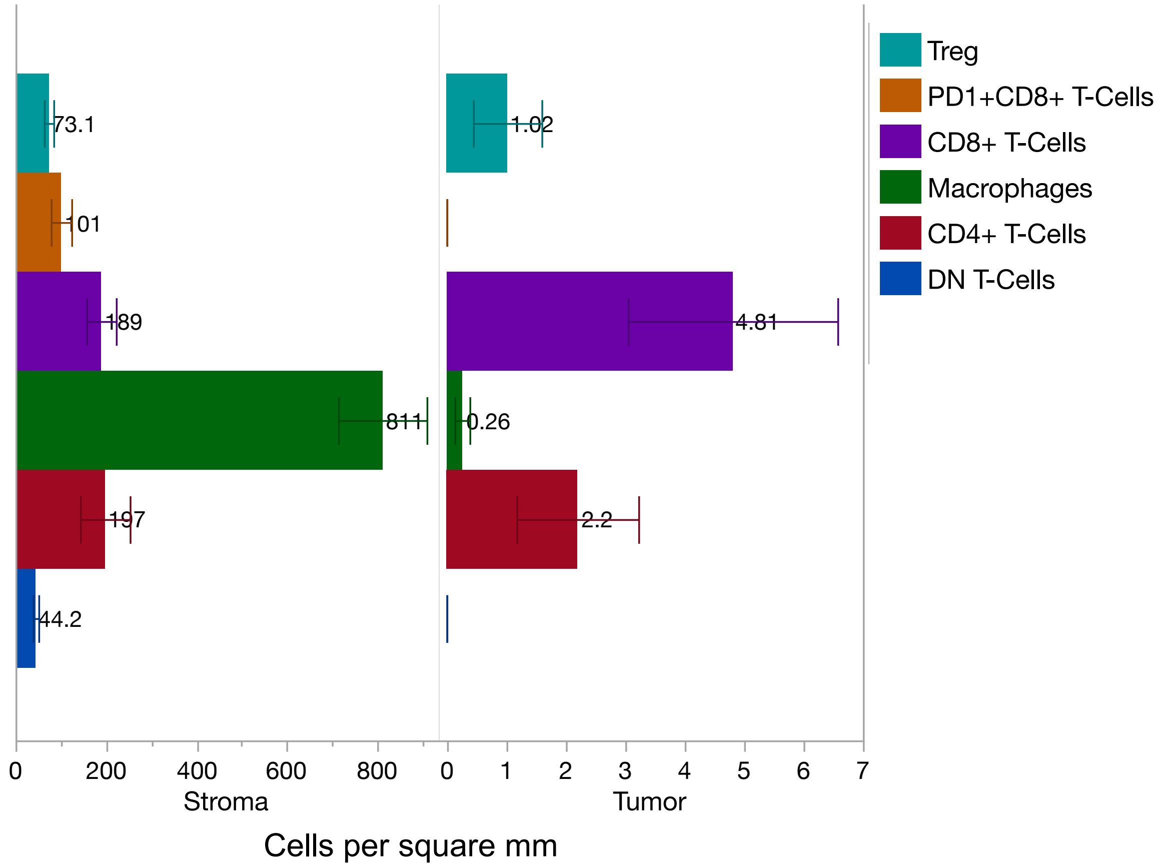

Figure S2: *Number of cells (per mm²) detected in the stroma and tumor area using the algorithmic tissue separation of the inForm® Tissue Analysis Software.*

Figure S3

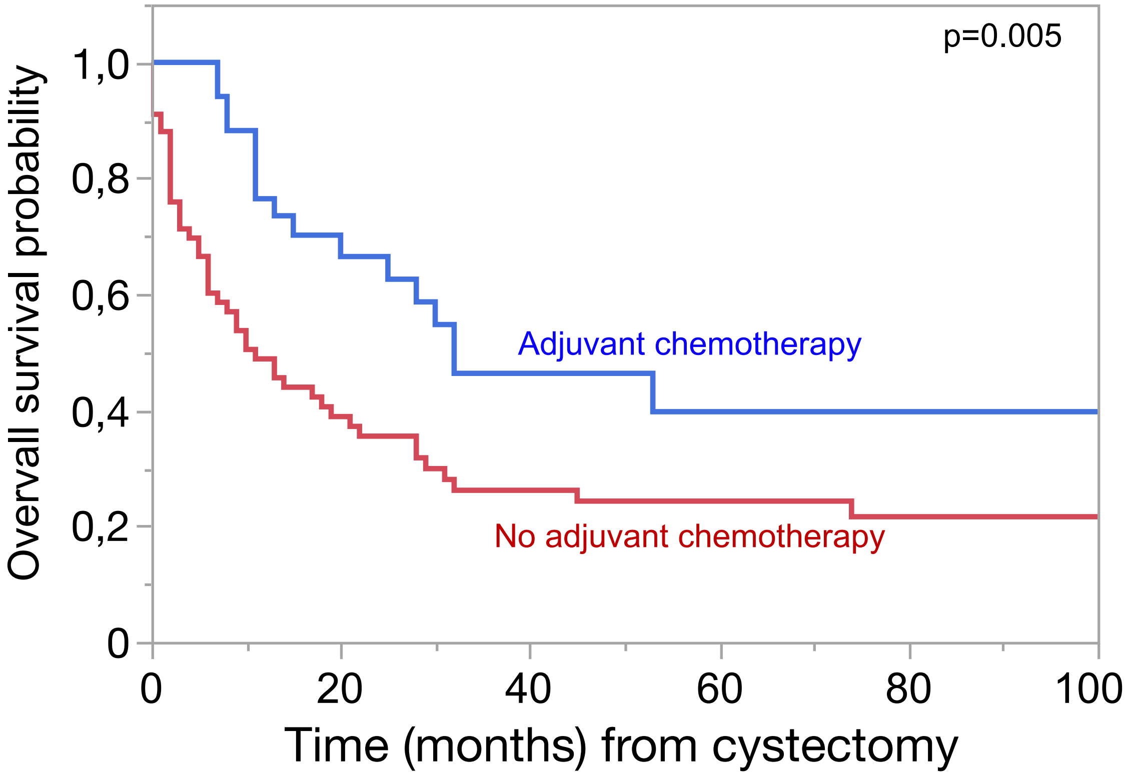

Figure S3: *Kaplan–Meier curves for overall survival probability for patients with (n=34) and without adjuvant chemotherapy (n=67); p(log-rank) = 0.005.*

Figure S4

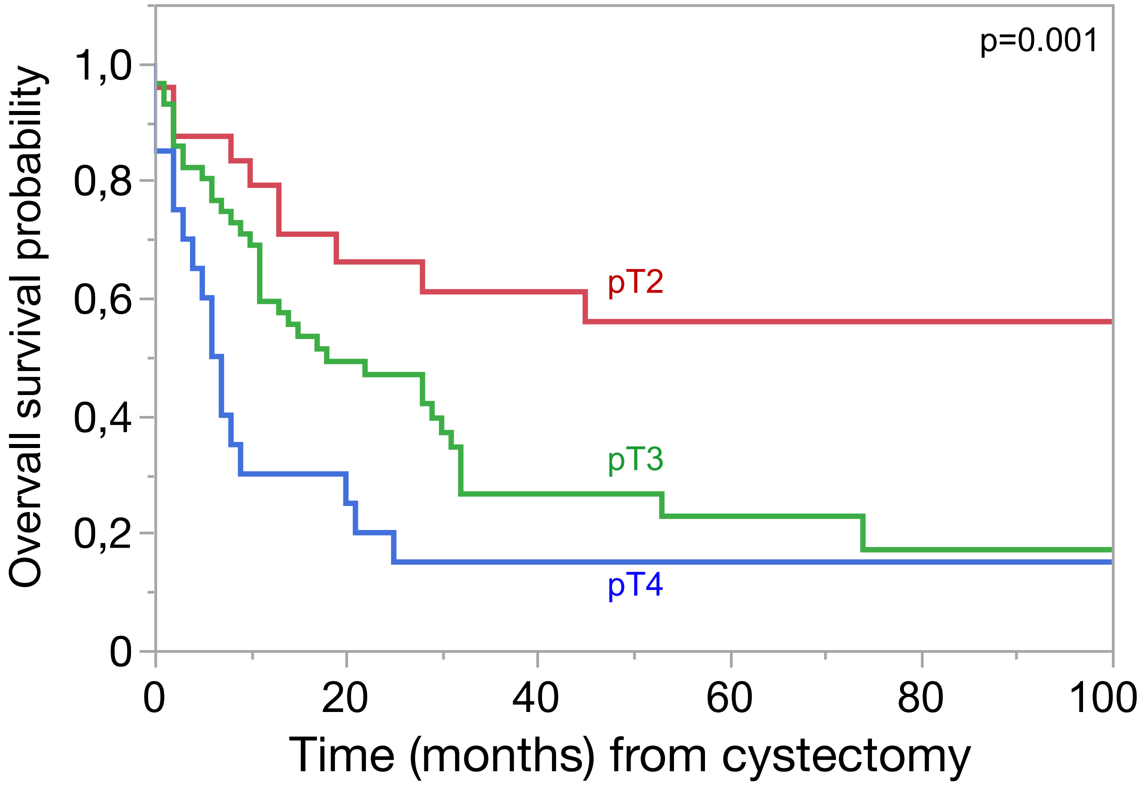

Figure S4: *Kaplan–Meier curves for overall survival probability stratified for patients by pathological tumor stage; p(log-rank) = 0.001.*

Figure S5

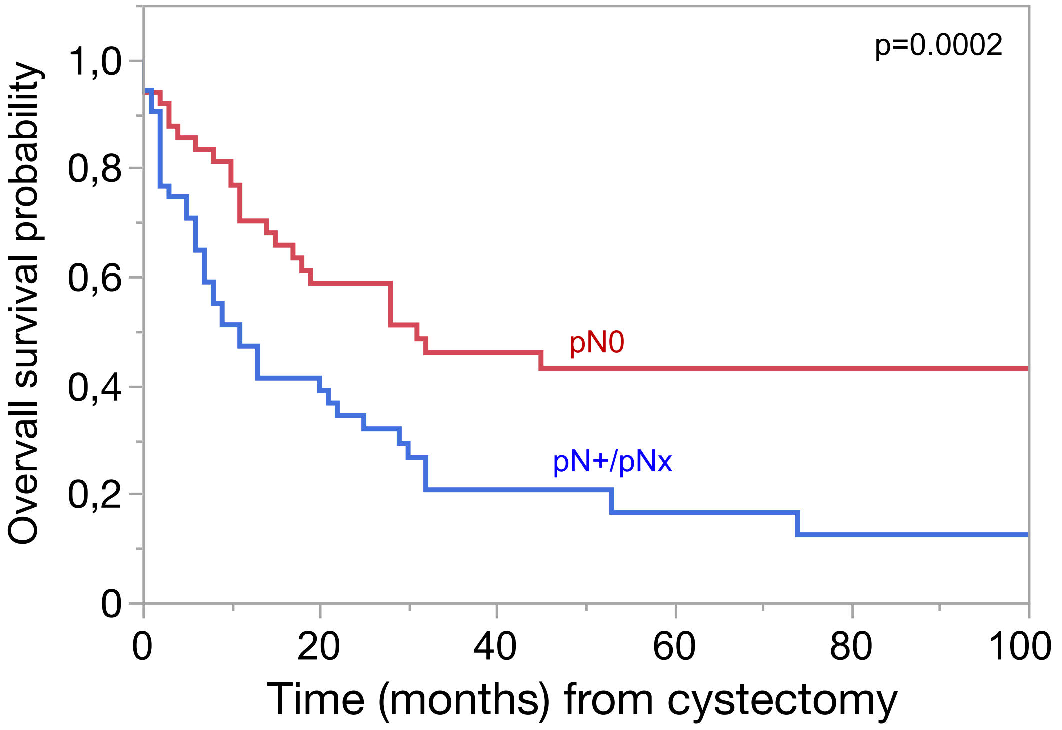

Figure S5: *Kaplan–Meier curves for overall survival probability stratified for patients by pathological lymph node stage; p(log-rank) = 0.0002.*

Figure S6

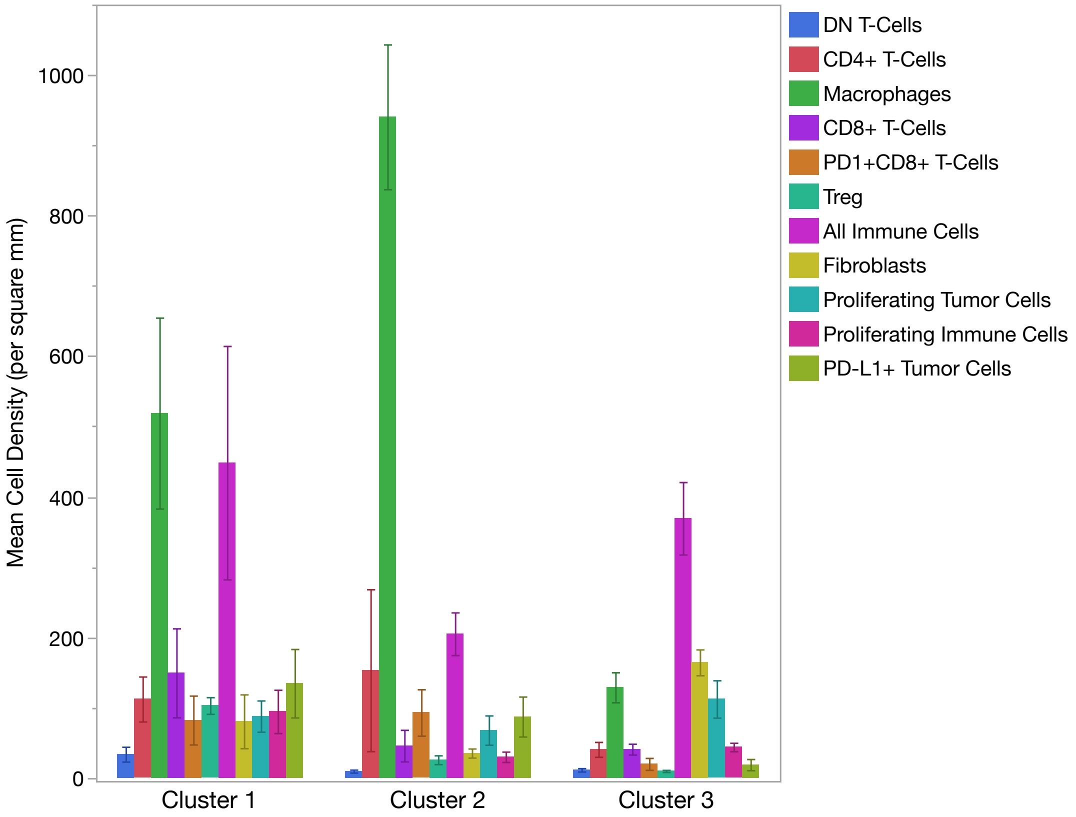

Figure S6: *Cell types (mean cell density/mm2) represented in the three clusters. Error bars show standard errors of means.*

Figure S7

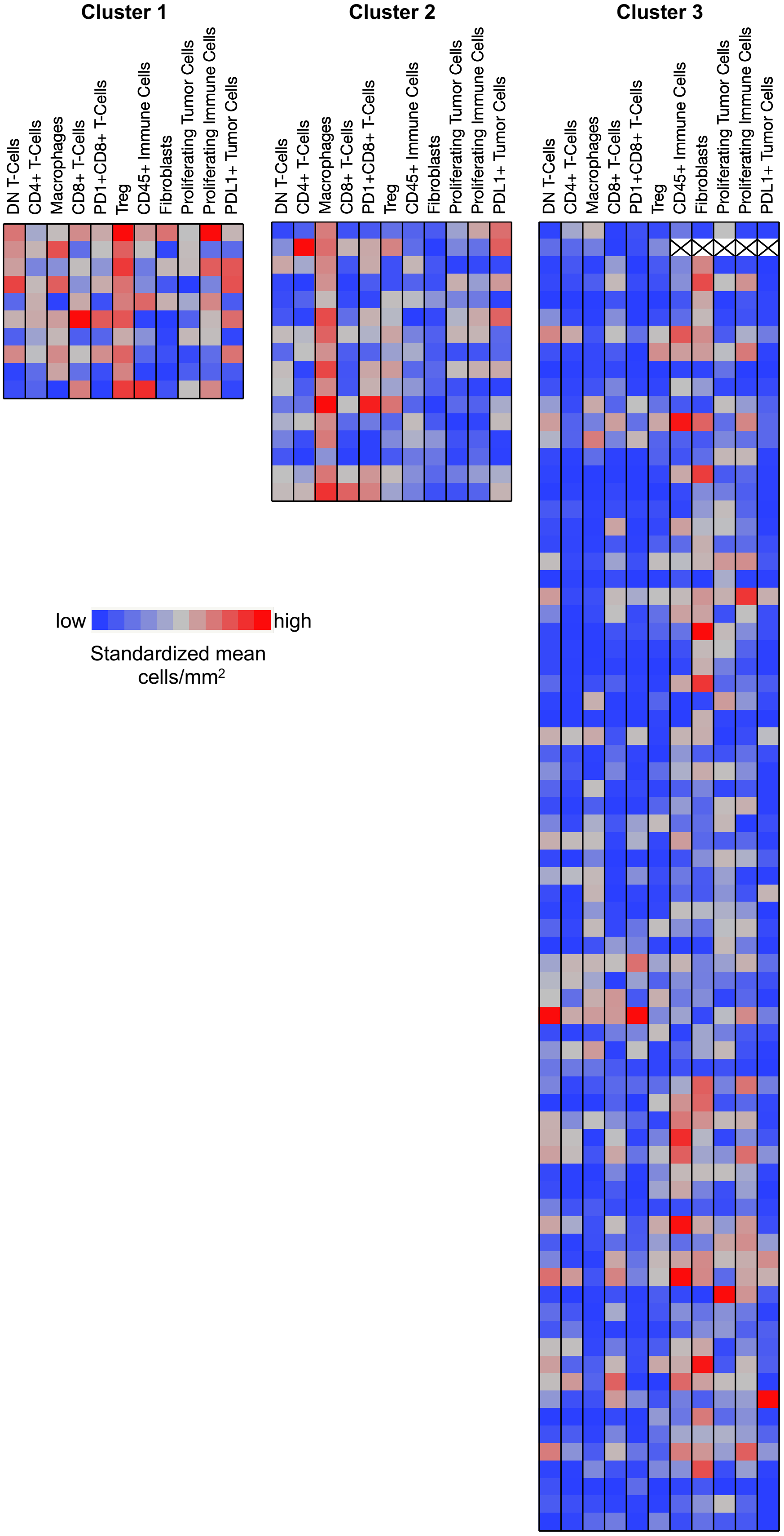

Figure S7: *Heatmap of Z-Scores (z = xi −mean(x)/ st.dev(x)) for each cell type and each sample.*

Figure S8

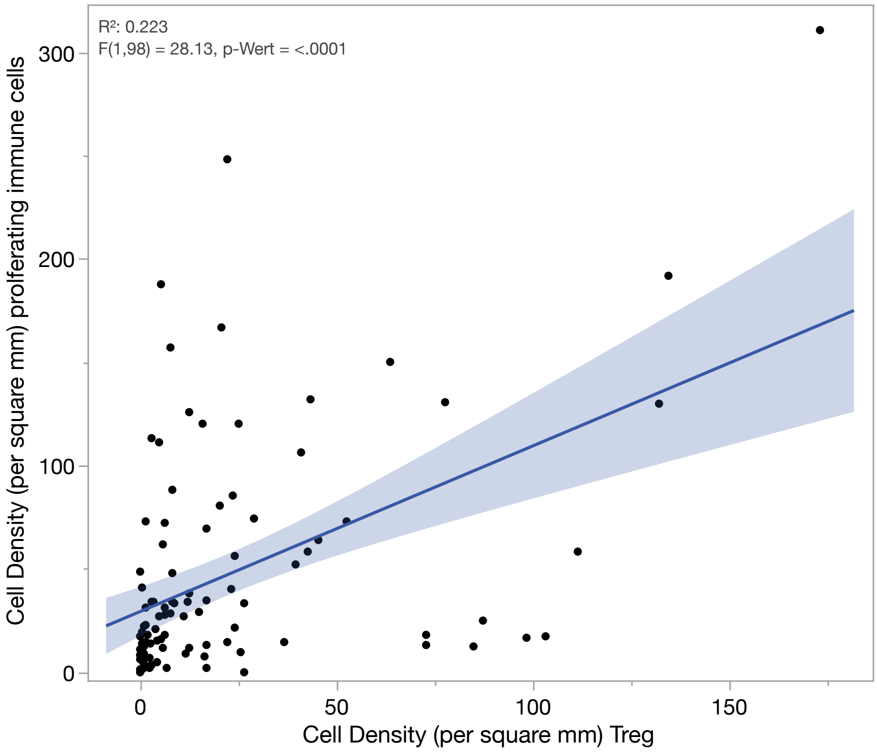

Figure S8: *Correlation plot of the cell density of Treg (FoxP3+) vs. proliferating immune cells (CD45+Ki67+).*

Figure S9

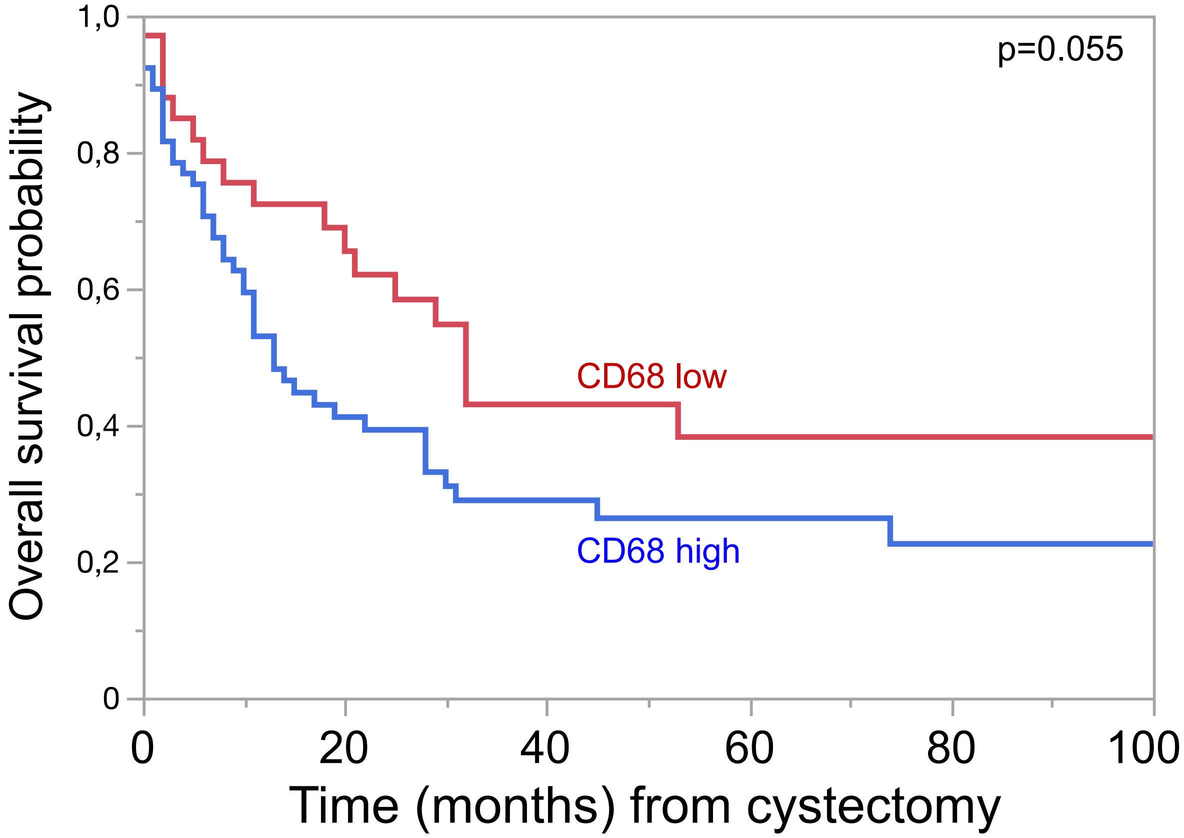

Figure S9: *Kaplan–Meier curves for overall survival probability stratified for patients by the macrophage marker CD68 with a cut-off 5%/all cells; p(log-rank) = 0.055.*

Figure S10

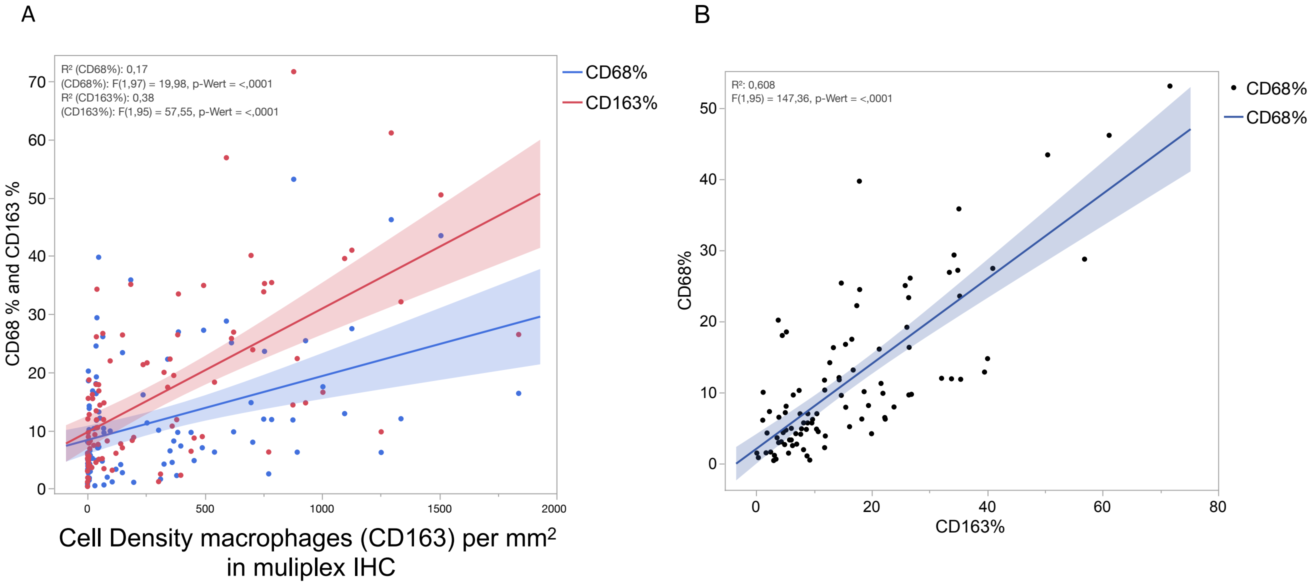

Figure S10: *Correlation plot of routine IHC for CD163 and CD68 vs. CD163 in the multiplex IHC panel (A). And Correlation between CD68 and CD163 expression in routine IHC (B).*
